# Visuo-Acoustic Stimulation’s Role in Synaptic Plasticity: A Review of the Literature

**DOI:** 10.3390/ijms221910783

**Published:** 2021-10-05

**Authors:** Emanuele Tonti, Mauro Budini, Enzo Maria Vingolo

**Affiliations:** Department of Biotechnology and Medical-Surgical Sciences, “Sapienza” University of Rome, 04100 Latina, Italy; tonti.oculista@gmail.com (E.T.); mauro.budini@uniroma1.it (M.B.)

**Keywords:** neuroplasticity, critical period, perceptual learning, acoustic and visual biofeedback, microperimetric biofeedback training

## Abstract

Brain plasticity is the capacity of cerebral neurons to change, structurally and functionally, in response to experiences. This is an essential property underlying the maturation of sensory functions, learning and memory processes, and brain repair in response to the occurrence of diseases and trauma. In this field, the visual system emerges as a paradigmatic research model, both for basic research studies and for translational investigations. The auditory system remains capable of reorganizing itself in response to different auditory stimulations or sensory organ modification. Acoustic biofeedback training can be an effective way to train patients with the central scotoma, who have poor fixation stability and poor visual acuity, in order to bring fixation on an eccentrical and healthy area of the retina: a pseudofovea. This review article is focused on the cellular and molecular mechanisms underlying retinal sensitivity changes and visual and auditory system plasticity.

## 1. Introduction

Neuroplasticity can be defined as the ability of the nervous system to modify its structure on the basis of different environmental changes and stimulation. This allows the brain to store and organize new information and to learn new skills. Furthermore, these processes are crucial in the growth and organization of sensory systems. The plasticity potential is higher during the development ages, and it is fundamental for the organism’s adaptation to the environment [1,2]. In fact, children are more prone to learn a new language, play an instrument, or practice a sport. At the same time, it is well known how a visual impairment in early life (caused by a congenital cataract, strabismus, etc.) can alter the development of visual pathways, causing amblyopia. Similarly, hearing impairment can lead to an abnormal auditory perception [2,3]. Even though neuroplasticity occurs mostly during childhood within a “critical period”, in adults, the nervous system maintains the ability to recover from brain injuries [3]. For example, in post-stroke patients, through task-specific training and physical exercise, it is possible to reactivate innate physiological and synaptic plasticity in order to perform an appropriate rehabilitation path [4]. Thus, the first evidence of neuroplasticity was given by studies on animals, first on animal tissue in vitro, then in vivo, and then more recently in humans as well. In fact, neuroplasticity mechanisms can be studied in vivo using functional magnetic resonance imaging (fMRI), electroencephalography (EEG), and transcranial magnetic stimulation (TMS) [4,5].

In this review, we summarize the concept of neuroplasticity and the mechanisms which drive the brain’s dynamic potential at the microscopic and macroscopic levels. Furthermore, we will discuss brain plasticity in visual and auditory systems, focusing on how their stimulation may affect neural plasticity itself and may be useful for therapeutic applications.

## 2. Neuroplasticity: Definition and Mechanisms

Neural plasticity can be distinguished as structural and functional plasticity. The structural plasticity refers to modifications occurring in neurons’ axons and dendrites in addition to renovation of these cells and synapses (neurogenesis and synaptogenesis). On the other hand, the functional plasticity comprehends the biochemical mechanisms behind synaptic efficacy [6]. This is a continuous remodeling process which allows short-term, medium-term, and long-term reshaping of the synaptic net, contributing to modifying or renewing its functions [7]. Thus, the brain’s plasticity plays a pivotal role throughout the lifespan of the individual, from the critical period in early development—with the creation of new neural maps thanks to learning through sense stimulations—to adulthood and old age, when these circuits are stabilized. However, this neural reshaping may also be elicited by peripheral or central nervous system (CNS) lesions [5,8]. For example, after a peripheral nervous lesion or limb amputation, the corresponding cortical areas begin to receive signals from peripheral areas surrounding the damaged site. This has been documented in the primary motor cortex (M1) after a peripheral nerve lesion, causing the extension of nearby cortical areas in the M1 territory [9]. On the other hand, if a cortical lesion occurs, its function ends up being carried out by nearby cortical areas. Furthermore, animal experimentations showed how, after an M1 ischemic lesion, the use of specific training, such as constraint therapy applied to the limb involved, could enhance the reorganization of unharmed M1 portions [10]. Merzenich et al. demonstrated how the primary cortical sensory map in adult animals can go through a process of reorganization after various peripheral sensory perturbations. In particular, studies on monkeys showed how after the section of the median nerve, somatosensory representation of its innervation areas undergoes rapid reorganization. Moreover, the cortical areas near the ones of the median nerve expand at its expense, as seen for the cortical representations of the ulnar nerve [11,12]. In a study of Kaas et al. based on macaques that underwent upper limb deafferentation 12 years earlier, the stimulation of the animal’s face produced activation of the area representing the deafferented limb, showing how there had been a reorganization of these two neighboring cortical areas [13].

### 2.1. Biological Basis of Neuroplasticity: Microscopic Aspects

The biological processes that underlie neuroplasticity take place at both the microscopic and macroscopic levels. Microscopic mechanisms include neurogenesis, synaptic activity modifications, reactivation of latent synaptic networks, and modulation of neural circuits mediated by glia and an extracellular matrix. During the early stages of development, the proliferation and differentiation of neurons and their structures (e.g., dendrites and axons), as well as their connections through synapses, take place [14]. Afterward, because of sense stimulation and experience, these networks are molded through apoptosis and the modification or regression of synaptic connections [15,16]. These structural changes may also take place after a brain injury, showing renovation in particular dendrites [17]. There is also evidence concerning the role of neurotrophins (NTs) in neural plasticity, mediating the differentiation and survival of neurons in synaptic transmission and reshaping [18,19]. In addition to neurogenesis, synaptic plasticity plays an important role in the brain’s reorganization. It refers to the modulation of synaptic efficacy due to repetitive nerve impulses. Thus, this process is based on changing the stimulation from a presynaptic to postsynaptic cell, providing an increase or decrease in synaptic efficacy, named long-term potentiation (LTP) and long-term depression (LTD), respectively [2,5,20,21]. LTP originates from rapid presynaptic depolarization of the synapses, which activates NMDA-type glutamate receptors in the postsynaptic membrane, causing a rise in intracellular Ca^++^ levels. This induces the expression of AMPA-type glutamate receptors in the postsynaptic membrane, leading to an increase in synaptic strength. It also causes the release of brain-derived neuronal growth factor (BDNF) in neurons, which enhances LTP and enlarges the dendrites ([5,22] “AMPARs and synaptic plasticity: The last 25 years”). On the contrary, LTD comes from slow repetitive stimulation of the synapses, which causes a migration of AMPA receptors in the cytoplasm [20]. While LTP has a key role in learning and memorization, as seen in the hippocampus, both LTP and LTD seem to mediate the reorganization of neural networks in the sensory motor cortex [23]. In a review article, Sheperd et al. described how *ARC* gene expression is involved in the regulation of synaptic plasticity. In fact, it seems to control the neural output of excitatory neurons by facilitating LTD and by modulating the expression of AMPA glutamate receptors [24,25]. In a work by Pfeiffer, B. and Huber, K., it is explained how, in order to maintain LTP and LTD as functional synaptic changes in the cortical areas, it seems that local or dendritic specific protein synthesis is required [26]. Another kind of synaptic plasticity is represented by the conversion of silent synapses in active connections. The organization of cortical networks in functional areas is granted by the activity of inhibitor GABA interneurons, which stop the horizontal connections between different areas. Events such as sensory deprivation or learning may interrupt this kind of control, unleashing these latent connections and creating a sort of short-term plasticity [27,28]. A synapse’s activity can also be directly influenced by neuroglia. This wide network, through the production of neurotransmitters and extracellular mediators, has the potential to improve synaptic transmission [29,30]. Moreover, glial cells can also communicate with each other by using gap junctions and intracellular messengers [31] to coordinate the activity of neural networks. Control of the neuronal activity is also accomplished by the extracellular matrix [32].

### 2.2. Biological Basis of Neuroplasticity: Macroscopic Aspects

Several mechanisms lead to functional reshaping at a macroscopic level. Macroscopic changes include cross-modal plasticity, a modality-specific brain area that is deprived of its usual sensory input and becomes responsive to the stimulation of other modalities. For example, the occipital cortex in visually impaired patients may be activated by sound changes [33]. Furthermore, a vicariation modality is possible, as the takeover of the function by areas not originally involved in the damaged performance that are remote from the site of the primary damage, known as diaschisis [34]. Other macroscopic mechanisms of plasticity are functional redundancies, or intrinsic reorganization of eloquent areas with multiple cortical representations of the same function within the same region. On the other hand, a reorganization within a functional network is another crucial pathway, as other regions belonging to the same functional network may be recruited: the perilesional areas first, and if still insufficient to the functional purpose, remote structures [8]. If the unimodal association areas are damaged, there is a rapid over-recruiting of new areas to sustain the impaired process based on an activation–hyperactivation pattern. These compensatory strategies were first described on the dorsolateral prefrontal or intraparietal cortices [35]. These mechanisms can also have a macroscopic impact on the volume of gray matter, as analyses revealed a region of increased gray matter in Broca’s area in the left inferior frontal gyrus in musicians, and studies of the anatomical effects after environmental experience and training in humans demonstrated a cortical volumetric increase [36,37].

## 3. Neuroplasticity in the Auditory System

The initial idea of plasticity applied to the hearing system was that of a process starting once the inner ear starts functioning and then shaped by experience only during a critical period in early development. However, in the last few decades, several studies demonstrated how the auditory system remains capable of reorganizing itself in response to different auditory stimulations or sensory organ modifications. Thus, the auditory system has a plasticity potential which also continues in adulthood. This process may vary from short-term adaptation to long-term modification of neural circuits, as seen in the case of hearing loss. This may derive from the effect of different sensory inputs (bottom-up processes) or the influence of learning, attention, or doing specific tasks (top-down processes) [38,39,40].

### 3.1. Mechanisms of Auditory Plasticity

An example of auditory system plasticity is stimulus-specific adaptation (SAA), a process leading to a lower neuron response to repeated acoustic stimulation [41]. This has been demonstrated by presenting a series of identical stimuli interspersed with individual different ones, during which neurons showed adaptation to first stimuli while they continued responding to the second ones. Such findings suggest how SSA could be a sort of adaptation of the auditory cortex to acoustic stimuli in order to focus only on relevant ones [39]. Hearing system plasticity may also happen as a consequence of hearing loss so that it reorganizes itself after the injury. The first studies about this kind of plasticity were performed on animals and adult humans after cochlear injury, analyzing changes in a neuron’s frequency sound selectivity and the remodeling of the corresponding area in cortical tonotopical map [39,42]. In fact, damage to hair cells in the cochlea or exposure to high-intensity sounds can result in the inability to discriminate precise sound frequencies. It has been noted that, after an injury, the portions of the auditory cortex surrounding the area which represented the damaged part of the cochlea expands at its expense [40,43]. The result is that a higher number of neurons focus on frequencies that are still heard. However, even if this plasticity may seem useful because it avoids losing frequency discrimination, it disrupts the neural coding in the auditory cortex. In fact, the reorganization of the auditory system, which has the aim to continue hearing certain frequencies after an injury, does not help to recover from hearing loss [33,39]. On the contrary, it seems to be a maladaptive process, as it has been associated with tinnitus [39]. This may result from the fact that, after an injury, some neurons in the auditory cortex start responding to different frequencies, which could lead to inappropriate coding of certain frequency stimuli [38,44,45]. Patients suffering from these kinds of injuries can benefit from the use of cochlear implants. In particular, it has been noted how hearing recovery largely depends on the auditory system plasticity induced by these hearing aids [46]. The role of these implants is to make the patient hear certain sound frequencies again after a cochlear injury so that the neurons which used to respond to those frequencies can be stimulated again. After implantation in deaf adults due to cochlear injuries, sound frequency discrimination and thus speech understanding are gradually restored [47,48]. These implants’ effects on the auditory cortex can be observed through magnetoencephalography (MEG). Pantev et al. reported increased evoked brain activity in the auditory cortexes of two deaf adults with implants, which related to the increase of neural activity in these areas due to the restored auditory stimuli [49]. It is important to consider that the beneficial effect of hearing aids depends on when they are implanted after the cochlear injury. We discussed above how, after an injury, auditory fields which were stimulated from that part of the damaged cochlea start responding to the frequencies of nearby neurons. This could lead to interference between these stimuli and those provided by hearing aids [38]. Moreover, the deprivation of this stimulation can also induce cross-modal plasticity of other sensory modalities at the expense of these auditory cortex areas [50]. The result is that these patients will not benefit from hearing aid stimulation, or at least these forms of plasticity can lead to a maladaptation if hearing aids are implanted late [51,52]. For this reason, in order to obtain the best result from hearing aids, it is important to consider early implantation after an injury [38,40].

Plasticity in the hearing system may also derive from the repetition of a specific pattern of tasks, consisting of focusing and discriminating specific auditory stimuli. This kind of training is known as perceptual learning. Most studies on this form of plasticity were based on training regarding sound frequency discrimination. During the training in animals, as the frequency identification improves, there is a progressive increase in the representation of those frequencies in the auditory cortex [53,54]. In humans, an example of perceptual learning is the one resulting from musical training, which influences the cortical processing of sound stimuli both in the auditory cortex and in the brainstem [55,56].

### 3.2. Biological Basis of Auditory Plasticity

Auditory plasticity develops at microscopic levels through different processes, including molecular and cellular mechanisms involved in cortex reorganization. Brain-derived neurotrophic factor (BDNF) plays a role in adult organization of the auditory cortex [57]. BDNF contributes to the maturation of GABAergic interneurons including parvalbumin (PV) interneurons, which contribute to regulating the onset and ending of critical periods and thus the plasticity potential [58]. In fact, a reduction of inhibitory transmission has the potential to re-open the plasticity window of the critical period [59]. This can be achieved through the loss of acoustic input at a juvenile age [60] or in adulthood [61]. Glutamate has also been found to have a role in auditory plasticity, as a block of NMDA receptors performed using ketamine reduces the amplitude and augments the latency of Mismatch Negativity (MMN). MMN is an evoked potential which occurs in response to an unexpected auditory stimulus, placed in a series of repetitive tones which differ from the latter. MMN is considered to be a measure of auditory plasticity, as it can be generated by the nervous system only if a memory trace of the repetitive tones has already been formed. In fact, the stimulus-specific adaptation (SSA) of auditory cortex neurons to these patterns seems to be necessary for MMN [62]. Microglia are also able to mediate cortical plasticity. These nonneural cells are activated after brain damage and act by removing neurons’ debris after their death, but they are also active in non-injured brains through monitoring synaptic functions and playing a role in their maturation or elimination through fagocitation of axonal terminals and dendritic spines. In a study conducted on mice, Paolicelli et al. showed these roles of microglia on the synapses. In particular, the study was based on mice lacking Cx3cr1, a receptor for fractaline, a chemochine which guides microglia migration. These receptors are usually expressed on neurons through which they recall the microglia. During their development, mice brains lacking these receptors showed lowered synaptic pruning, which resulted in immature synapses [63,64]. Microglia activity may be induced by traumatic noise exposure [65]. Neurotransmitters have the potential to influence synaptic plasticity. Cholinergic projections to the auditory cortex can play a role in plasticity by inhibiting PV interneurons during the development ages in the critical period [66] and also in adulthood [67]. In an experiment on cats, McKenna et al. showed how, in the presence of ACh during a presentation of a series of different tones with different sound frequency stimuli, there was a facilitated response in the auditory cortex toward frequencies which differed from the neurons’ “best frequency” [68]. Kilgard et al. demonstrated how a simultaneous electrical stimulation of an adult rat’s basal forebrain (thus with consequent cholinergic cortical projections) and presentation of auditory stimuli with a precise frequency produced an expansion of the auditory cortex tuned on that frequency [69].

## 4. Neuroplasticity in the Visual System

As for the auditory system, the idea of plasticity of the visual cortex strictly limited to the early stages of development has gradually changed in the last few decades. Traditionally, vision has been the system of choice for exploring the effects of sensory experience on cortical plasticity, particularly during development. Some well-known models of plasticity in the visual cortex derive from impaired visual experience in early life due to strabismus, anisometropia, or congenital cataracts. Therefore, these conditions can cause amblyopia, which is a unilateral reduction of the best-corrected visual acuity that persists during the patient’s life [3,70]. This is the result of ocular dominance of the eye with better visual acuity, as the vision impairment affecting the other eye results in visual deprivation of its stimuli. The age at which the visual deprivation takes place influences the grade of plasticity of the visual system and therefore the potential recovery of vision if the appropriate treatment is applied [71,72]. Studies about visual impairment in the development ages in humans, as well as experiments of visual deprivation in animals, led to the concept of a critical period, within which the plastic potential of the visual cortex is at its highest levels, developing in the early stages of life [72,73,74]. At the same time, after this period, there is no chance of any further plasticity of the visual system and therefore no visual recovery potential. During the critical period, this impaired ocular dominance can be treated with excellent results by occluding the non-affected eye for a prolonged time [75,76]. However, recent evidence in the literature showed how there is a chance of treating this imbalanced ocular dominance, suggesting that a plasticity potential may also be elicited in adulthood. In fact, new treatment approaches for amblyopia in adults started, including perceptual learning exercises and the use of video games [77,78]. More generally, the plasticity potential after the critical period has also been shown in studies of animals through pharmacological treatment, sensory-motor stimulation (known as environmental enrichment), and physical activity [79,80,81]. Moreover, neural plasticity in the visual cortical areas (V3A and V7) has also been found in healthy adults after training based on juggling, which is an activity that involves visual and motor system integration [82].

### 4.1. Mechanisms of Visual Neuroplasticity

Neuroplasticity in the visual system takes place through various mechanisms, including perceptual learning and adaptation [1].

Perceptual learning, as described above for the auditory system, consists of practicing specific visual tasks, leading to increased ability in visual stimuli discrimination. The training can be based on asking participants to identify specific textures or assess the orientations of various visual stimuli [83]. The effects on the visual system have been observed in the visual cortex through fMRI by analyzing the consequential increase of the blood oxygenation level-dependent (BOLD) signal in the primary visual cortex, which can be considered a sign of plasticity induced by perceptual learning [70,84]. A more complex form of perceptual learning is the one induced by playing video games, which provides a stimulation of different senses, improving perception, spatial selective attention, and visuo-motor coordination, inducing plastic changes in different areas of the brain [85,86,87]. As was previously mentioned, video games also provide an increase in visual acuity, spatial attention, and stereoacuity in amblyopic patients [88]. Another form of visual plasticity is the one known as stimulus-selective response potentiation (SRP), which consists of an increased response of the visual cortex to repetitive visual stimuli, measured as augmentation of the visual evoked potentials (VEPs). This phenomenon is thought to be the mechanism on which perceptual learning is based [89].

Adaptation can be considered an adjustment in the neuronal activity of the visual system when looking at a pattern of visual stimuli, inducing changes in sensitivity and, thus, the appearance of other patterns [1,88]. We can consider forms of short-term adaptation, like light adaptation, so that the neural response to a light source is influenced by repetitive exposure to that stimulus and also by the ambient lighting conditions [88,90,91]. On the other hand, there are also forms of long-term adaptation to color vision and blurred images (defocus, like in myopia and hypermetropia) which take place over months or years. For example, after cataract surgery, the return to a normal color appearance may take some months [88]. Other forms of adaptation may result from eye injuries or even changing a glasses prescription. In fact, there is potential adaptation to a long-term refractive error, such as myopia or hypermetropia, which leads to better visual acuity [92].

### 4.2. Biological Basis of Visual Neuroplasticity

There are several biological processes that underlie visual plasticity. We can distinguish functional plasticity, which derives from synapse activity and efficiency, and structural plasticity, involving morphological modifications in neurons, neurogenesis, and the creation or disruption of new synapses and dendrites [1,6].

It is known that the maturation of GABA-mediated intracortical inhibitory connections plays a pivotal role in the onset and closure of the critical period for the visual system [93,94]. Indeed, a study on animals with monocular deprivation but also lacking a GABA-synthesizing enzyme resulted in no ocular domination of the non-occluded eye [95]. On the other hand, a study about the reduction of GABAergic transmission in adult rats led to a reactivation of the visual cortex’s plasticity, even after monocular deprivation [96]. Numerous neurotransmitters demonstrated a plasticity potential in the visual cortex. The use of serotonin reuptake inhibitors shows an improvement in plasticity, as well as acetylcholine. On the contrary, a block of dopamine receptors and calcium channels results in lower plasticity [97]. ACh can modulate neural responses in the visual cortex. In particular, in a cat’s visual cortex, ACh provides a higher response to visual stimulation, enhancing the selectivity to the precise orientation or direction of visual stimuli [98]. As was described for the auditory cortex, the stimulation of the basal forebrain at the reticular formation results in an increase of evoked potential in the visual cortex [99]. The cholinergic system may also play a role in perceptual learning in the visual system. In fact, when associated with specific visual training or with a stimulus-enriched environment, ACh can improve the synaptic strength and exert a reorganization in the visual cortex. Chamoun et al. demonstrated that through cholinergic activation either using electrical or pharmacological stimulation, combined with visual stimulation, the visual evoked potentials, visual acuity, and visual cortex neurons’ responsiveness in adult cats can improve [100]. Recent findings demonstrated how a reactivation of plasticity in the adult visual cortex can be mediated by the use of histone deacetylase inhibitors, showing how the chromatin structure in the central nervous system can modulate plasticity [101]. Moreover, another factor capable of influencing plasticity is CREB, a transcription factor whose level rises after monocular deprivation but declines gradually with the maturation of the visual cortex [102]. Furthermore, many studies demonstrated how mitochondrial activity can regulate synaptic activity, as well as the morphological reorganization of dendrites and synapses [103,104]. In particular, after molecular manipulation to reduce the dendrite’s mitochondria, a reduction of synapses and dendritic spines is observed. On the contrary, an augmentation of these mitochondria acts as a promoter of synaptic plasticity [104].

Regarding structural plasticity, it has been shown that in animals exposed to an environment enriched with visual stimuli, such as objects disposed in their cages, an improvement in the cortical thickness in the occipital region has been noticed. In particular, several studies showed how this can lead to an increase in the number of dendritic spines and synapses [105]. In the same way, structural modifications in cortical organization have been found in humans during perceptual learning activities, such as language studies, juggling, and physical exercise [82,106].

### 4.3. Visual Plasticity in Retinal Degenerative Diseases

A valid model of study of neural plasticity in the visual system is given by some retinal degenerative diseases, such as retinitis pigmentosa (RP) and age-related macular degeneration (AMD).

Retinitis pigmentosa (RP), which is a hereditary disease, consists of progressive dysfunction of the photoreceptors, starting from the rod cells in the periphery and then involving the cone cells in the central retina [107]. Recent studies on these patients using fMRI showed how the region of the visual cortex deprived from retinal stimuli after the retinal degeneration of RP (named the lesion projection zone (LPZ)), can be excited by doing specific visual tasks. On the contrary, during passive visual stimulation, no activation of the LPZ was observed. To explain this, the authors hypothesized that the lack of retinal input to the LPZ could have reactivated preexisting extrastriate feedback signals, which are masked in the healthy retina. This could be considered a form of plasticity, even though the authors excluded a wider rewiring of the visual cortex [108]. Other studies demonstrated a form of cross-modal plasticity, leading to visual cortex activation during a tactile task while the patients suffering from RD were blindfolded. Another study found cortical plasticity in RD patients by analyzing both retinal (multi-focal ERG) and cortical (multi-focal VEP) functioning. In particular, they found a direct correlation between focal retinal functional loss and functional reorganization in the visual cortex [109]. However, other studies did not find any sign of visual cortex reorganization in RD [110,111].

When talking about age-related macular degeneration (AMD), in this case, the disease affects the center of the retina, which leads to progressive central vision loss. As a result of this, AMD patients use for fixation a peripheral retinal area acting as a pseudo-fovea (named preferred retinal locus (PRL)) [112,113], which can be chosen autonomously by the patient even before starting rehabilitation procedures, such as using microperimetry rehabilitation training (MRT). Some studies stated that this adaptation derives from a rewiring of the primary visual cortex, since the neurons in the LPZ start responding to stimuli from retinal areas neighboring the retinal degeneration, which correspond to the PRL [112,114]. In an fMRI study, the authors observed how there was LPZ activation after stimulation of not just the PRL but other paracentral areas [113,115], even if the PRL had a wider representation in the visual cortex [116]. Another study conducted on adult cats and monkeys with bilateral lesions of the central retina showed that by stimulating retinal areas near the lesion, reorganization of the cortical retinotopic map was noticeable. In particular, the rewiring of the visual cortex took place after months, showing a shift of its representation for the portion of the retina near the lesion [117]. On the other hand, like for RP, there was no clear evidence in some studies of visual cortex plasticity in AMD patients [118,119].

In conclusion, the amplitude of the visual cortex plasticity is not yet well-established. The results of the studies that we reported here show discrepancies about the results regarding visual system rewiring. This could be due to differences in the visual tasks used to study the plasticity phenomenon, as well as the fact that there was no homogeneity between the studies’ subjects regarding the stages of disease and due to the limited number of subjects. However, it is clear how a certain grade of plasticity can also be present in adulthood [1,120].

## 5. Microperimetric Biofeedback Training and Plasticity of the Visual Cortex

One of the noblest rehabilitation strategies in ophthalmology is Microperimetric Biofeedback Training (MBT). It consists of reeducating the visual system to a new visual condition, promoting retina–brain transmission and thus cortical plasticity [121].

Based on the fact that patients with absolute central scotoma develop a self-adaptation by directing fixation to the eccentric areas of the retina, known as the preferred retinal locus (PRL) [112,113,122], rehabilitation efforts aim to strengthen the fixation stability in that region or even help patients to choose a more efficient PRL with a higher retinal sensitivity in order to improve visual function in terms of visual acuity, reading speed, etc. [123]. Moreover, the fixation stability is usually altered in patients with central vision loss, using a larger retinal area for fixation when asked to fixate on a target that is away from the primary position gaze. The biofeedback approach improves the fixation stability by instructing patients to move their eyes according to audio feedback into a desired final fixation position [124]. There are many diseases for which treatment may include the MBT, such as age-related macular degeneration (AMD), advanced glaucoma, myopia, Stargardt disease, choroidal neovascularization (CNV), and a macular hole after vitreoretinal surgery.

Treatment for large macular holes (LMHs) is based on vitreoretinal surgery, but even though most patients reach the anatomical result of the hole’s closure, the functional outcome in terms of visual acuity recovery is not always as good as expected [125]. In a study on patients who underwent surgery for closure of large macular holes (LMHs), Sborgia et al. showed how after MBT training sessions using light stimulus and an acoustic tone in the first group, there was significant improvement in macular and central macular sensitivity and in fixation compared with the control group excluded from the training [126]. The group of Maneschg et al. was able to improve the fixation stability in patients which suffered with impaired fixation even after the surgical treatment for that condition [127].

**Figure 1:**
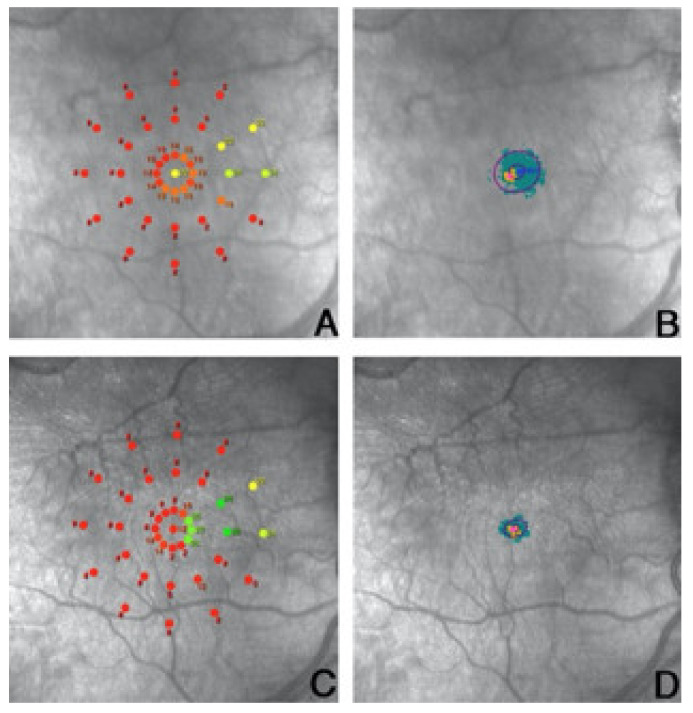
(**A**) Retinal sensitivity map of the RP patient and (**B**) fixation behavior of the patient, here represented by the bivariate contour ellipse area (BCEA), both prior to the training session. (**C**) Retinal sensitivity fixation map and (**D**) fixation behavior represented by the BCEA after the training session was completed. We can see here a substantial increase in the patient’s fixation behavior, which correlates with best corrected visual acuity (BCVA) improvement and with patient satisfaction after the training session. Images scale bar 300 micron.

AMD is a classic application of this therapeutic option and highlights the physiological process of using areas outside the damaged fovea: the above-mentioned PRL. The PRL is defined as an area that contains the center of a target image for over 20% of a fixation interval. The PRL, which is single in its physiological conditions, can become multiple if the scotomatous area increases [122,128]. On its side, the nervous system, with cortical neurons located in the retinotopic position corresponding to the damaged retinal area, does not receive direct stimulation from the damaged region, having less activation than other cortical neurons. However, those neurons start receiving small activity originating from non-altered neurons nearby, these connections are gradually reinforced, and the system finally evolves to a new stable state [129,130]. In order to improve the fixation to the most efficient PRL for the patient, microperimetric training may adopt both visual biofeedback and auditory feedback. In a study on late-stage AMD patients, Vingolo et al. compared the efficacy of acoustic biofeedback with visual feedback consisting of a flickering checkerboard pattern. The results showed that both biofeedback types were effective in the treatment of AMD by improving the visual acuity, fixation, reading speed, and life quality of the patients. Moreover, the rehabilitation granted an improvement in retinal sensitivity. Authors, starting from other studies’ conclusions, hypothesized a pivotal role from visual cortical plasticity in the form of reorganization of visual receptive fields in V1, as well as a possible functional reorganization of the retinal layers so that residual retinal cells could act as a substitute of photoreceptors and the outer retinal layers [123,131]. Some authors concluded how audio biofeedback can increase the patient’s attention, improving his or her performance in maintaining the fixation on the chosen PRL. Furthermore, they hypothesized that such acoustic stimulation may facilitate communication between intraretinal neurons as well as retina–brain transmission, supporting a “remapping phenomenon” [132,133]. In another study, Vingolo et al. compared two different types of acoustic biofeedback in a single group of 30 wet-AMD patients by using the MAIA2 Vision Training Module. In a first session, the patients were guided during rehabilitation by a standard acoustic biofeedback used in MBT. Then, after a washout of 3 months, they underwent the same rehabilitation schedule but used Mozart’s “Sonata for Two Pianos” as acoustic biofeedback to guide the fixation to the proper PRL. The results showed how the visual acuity, fixation stability, reading speed, and retinal sensitivity outcomes had significant improvement after the second session of training (Figure 1).

Moreover, patients stated that maintaining fixation on the target PRL was easier with Mozart’s music piece [134].

In a work by Ratra et. al based on patients with irreversible central scotoma deriving from different pathologies (such as AMD, CNV, Stargardt disease, traumatic macular scars, and myopic degeneration) demonstrated how the patients could benefit from an improvement in fixation, visual acuity, and macular sensitivity. The recovery was better in younger patients and in those with smaller scotoma sizes [135].

There are also other kinds of training protocols used in MBT. For example, a study from Maniglia et al. used MBT to treat patients with macular degeneration. The training was based on contrast detection and a lateral masking configuration. This rehabilitation protocol allowed for long-lasting improvements in visual acuity and contrast sensitivity [136].

## 6. Conclusions

There are different forms of plasticity that remain widely functional in the adult visual system, as proven by perceptual learning and long-term adaptation. Numerous biological systems are involved in the interaction of functional and structural plasticity. Perceptual optimization depends on changes in the environment and in the observer that are likely to involve different forms of plasticity that act together. In the future, efforts must be directed toward increasing understanding of these mechanisms to reach new forms of diagnosis and treatment of ophthalmic disorders, such as amblyopia, rehabilitation after severe retinal disorders, and the improvement of surgical outcomes after cataract and refractive surgery.

## Data Availability

Not applicable.
